# Acute Inferior ST-Elevation Myocardial Infarction Due to Wraparound Left Anterior Descending Artery

**DOI:** 10.7759/cureus.18701

**Published:** 2021-10-12

**Authors:** Anwar Nabeel Jafri, Mohammad Ali Salem Al Shehri, Mohammad Ahmed Anwar, Mohammad Said Abdelmonem

**Affiliations:** 1 Cardiology, Armed Forces Hospital Southern Region, Khamis Mushait, SAU

**Keywords:** major st elevation, mimicking st elevation, wrap around lad, lad occlusion, inferior st elevation

## Abstract

Most of the inferior ST-elevation myocardial infarctions (STEMIs) are due to the occlusion of the dominant right coronary artery, but there are few exceptions. In order to diagnose the actual life-threatening STEMI, we should be aware of the exceptional causes. Here, we present a case of a 69-year-old female with the first diagnostic electrocardiogram report interpreted as inferior STEMI, but the culprit occlusion was later found to be by left anterior descending artery in coronary angiography. All these observed circumstances are reported accordingly.

## Introduction

Acute occlusion of the right coronary artery (RCA) represents ST elevation in inferior leads in the electrocardiogram, while acute occlusion of the left anterior descending (LAD) coronary artery represents precordial leads [1]. Early recognition of this life-threatening disease is vital [2]. There should be reminders for the rarest cause and appearance on electrocardiograph lead to differences in angiographic findings. There are few cases reported as differences on ECG and angiographic culprit vessels involved. So, in our setting, this is the first seen case due to wrapped around LAD.

## Case presentation

**Figure 1:**
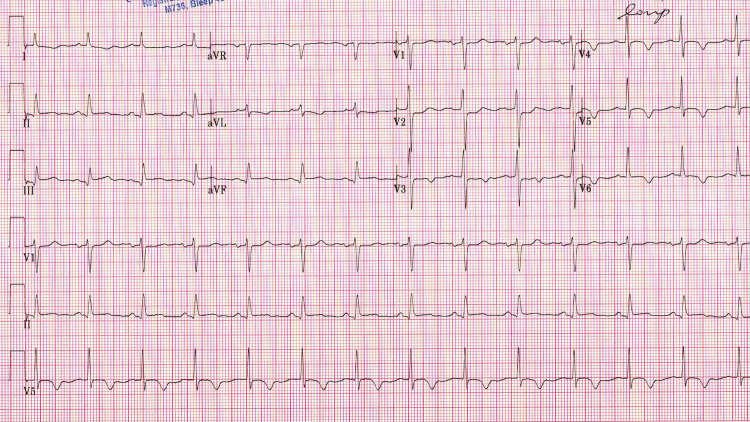
Electrocardiogram on admission showing inferior ST elevation in lead II, III, aVF and T wave inversion in lead V3- V6. aVF: augmented vector foot

A 69-year-old woman was presented to our hospital with a complaint of resting central chest pain which was non-radiating and of increasing in intensity. She was hypertensive and had diabetes for 10 years. On physical examination, her vitals were reported normal (pulse: 100/min, blood pressure {BP}: 140/90 mmHg, O_2_ saturation: 92% on room air), there were no murmur on cardiac examination but bilateral basal rales on the chest examination. An electrocardiogram on presentation demonstrated ST-segment elevation in the inferior leads (II, III, augmented vector foot {aVF}) and T wave inversion in V3-V6 (Figure 1).

On transthoracic echocardiogram (TTE), there was marked impairment of global systolic function and grade III diastolic dysfunction with segmental wall motion abnormalities in mid-anterior and inferior segments.

**Figure 2:**
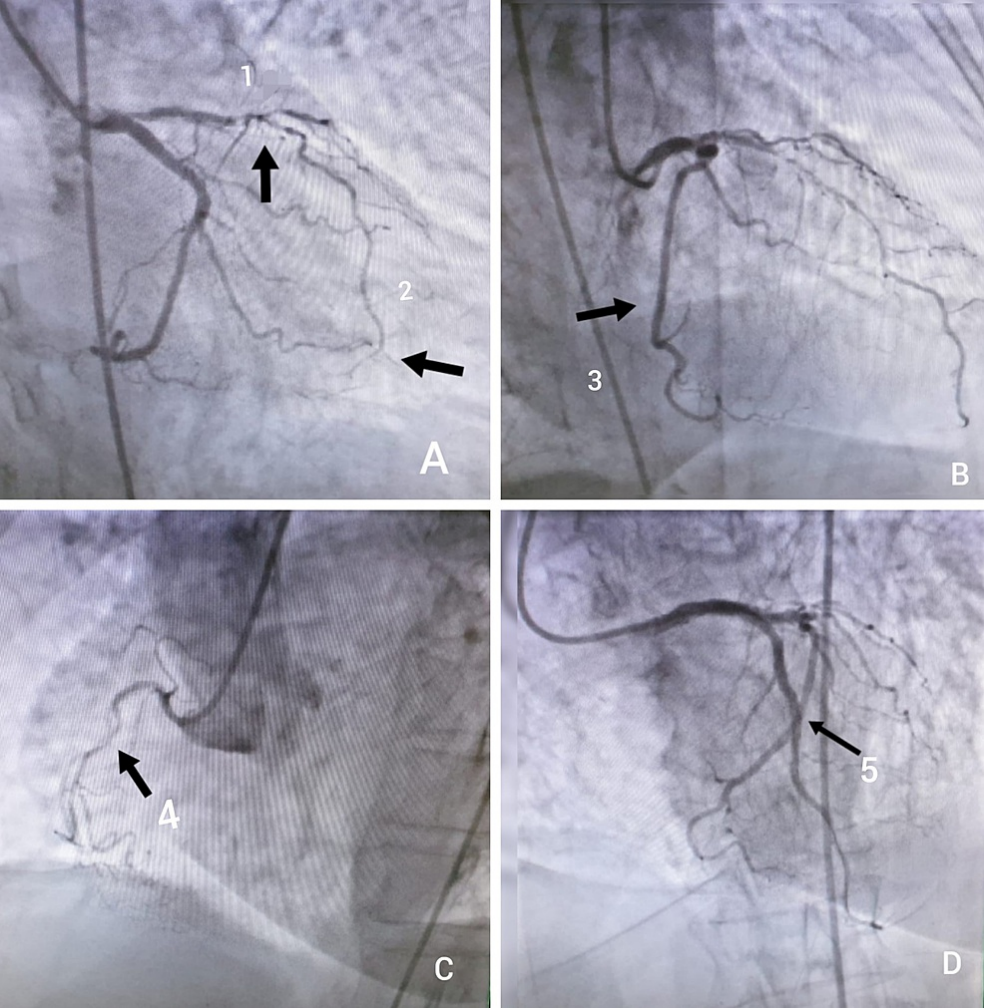
Coronary angiogram shows ostial LAD lesion, wraparound LAD artery, and post CAG to LAD. (A) - 1 = ostial LAD lesion, 2 = wraparound LAD artery; (B) - 3 = LCx artery; (C) - 4 = non-dominant RCA; (D) post CAG to LAD (culprit  vessel) LAD: left anterior descending; LCx: left  circumflex; RCA: right coronary artery; CAG: coronary angiography

Blood markers for infarction had been done which were quite high (hs-troponin-I: 1344 pg/ml). She was initially managed in the emergency room with antiplatelet and IV heparin and shifted to the cath lab. The door to balloon time was within 30 minutes. Coronary angiogram showed left dominant system. Left main stem was short and normal. Left anterior descending artery had ostial narrowing of 90% while the proximal to mid-segment showed 99% narrowing. Left circumflex artery was the dominant vessel with no significant disease. Right coronary artery was non-dominant and showed 80% proximal stenosis, and percutaneous coronary intervention (PCI) had been done to LAD (Figure 2).

The patient stayed in the coronary care unit for further stabilization and was discharged with a good general health condition.

## Discussion

The unusual course of coronary artery may be the cause for increased risk of sudden death, angina pectoris, heart failure, and arrhythmias [3]. A twelve-lead electrocardiogram (ECG) is still the reliable standard for diagnosing the size and site of infarction. Decisions on the basis of initial investigation may save time and facilitate the management and prevention of emergencies [4,5].

Inferior STEMI correlates with the occlusion of all three coronary arteries; 80% of inferior STEMI are because of dominant right coronary artery, while dominant left circumflex artery is only involved 18% of the time and rarely, there is wraparound left anterior descending artery causing inferior STEMI [6]. As the occlusion of LAD artery showing STEMI in the inferior lead and indicated anterior myocardial infarction is an unusual presentation of ST-segment elevation. It is nearly impossible to diagnose a wraparound LAD diagnosed by ECG. This anatomic identification is clinically substantial because of the higher long- and short-term mortality and morbidity [7,8].

There is a case report on the ECG with wrapped around LAD supplying the inferior left ventricle and showed acute anterior myocardial ischemia similar to our report [9]. Tamura et al. found out that inferior STEMIs were observed only in 12 patients with both a distal LAD occlusion and wraparound LAD [10].

## Conclusions

Due to the different collateral supplies and demand relationship, there is often no smooth extent of infarction in related vessel territory. Wraparound LAD anatomy is now presenting with ST-elevation, so there is a need for further case series and research articles to find out the significance, benefits, and mortalities associated with it. Such anomaly can have a significant impact on the outcome which the cardiologist should be aware of and reminded of.
